# The Importance of Pericytes in Healing: Wounds and other Pathologies

**DOI:** 10.3390/ijms18061129

**Published:** 2017-05-24

**Authors:** Hannah M. Thomas, Allison J. Cowin, Stuart J. Mills

**Affiliations:** 1 Centre for Regenerative Medicine, Future Industries Institute, University of South Australia, Mawson Lakes 5095, Australia; hannah.thomas@mymail.unisa.edu.au (H.M.T.); allison.cowin@unisa.edu.au (A.J.C.); 2 School of Pharmacy and Medical Sciences, University of South Australia, Adelaide 5000, Australia; 3 Cooperative Research Centre for Cell Therapy Manufacturing, North Terrace, Adelaide 5000, Australia

**Keywords:** pericyte, MSC, wound healing, cell therapy

## Abstract

Much of current research investigates the beneficial properties of mesenchymal stem cells (MSCs) as a treatment for wounds and other forms of injury. In this review, we bring attention to and discuss the role of the pericyte, a cell type which shares much of the differentiation potential and regenerative properties of the MSC as well as specific roles in the regulation of angiogenesis, inflammation and fibrosis. Pericytes have been identified as dysfunctional or depleted in many disease states, and observing the outcomes of pericyte perturbation in models of disease and wound healing informs our understanding of overall pericyte function and identifies these cells as an important target in the development of therapies to encourage healing.

## 1. An Introduction to Pericytes

### 1.1. Morphology, Location and Function

**Figure 1:**
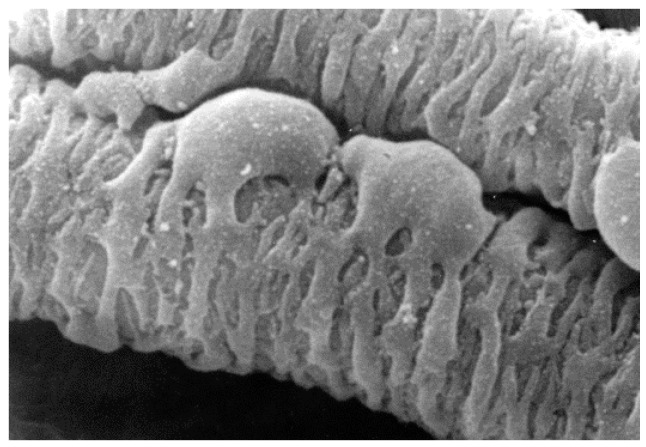
SEM showing pericyte processes spanning a venous capillary of the rete mirabile from an eel swimbladder. Image was kindly supplied by Professor Roger C. Wagner, University of Delaware.

Pericytes are cells found on the outside of blood vessels [1]. Their long processes encircle the abluminal surface of those vessels and attribute structural integrity to the vessel wall. Pericyte morphology is characterised by minimal cytoplasm, a prominent nucleus and projecting processes which wrap around associated capillaries (Figure 1) [2].

From their position on the outer surface of the blood vessel, pericytes interact with endothelial cells (ECs), which reside on the other side of the basement membrane, through adhesion plaques which provide adherence between the ECs and the pericytes. Peg-and-socket contacts facilitate the diffusion of molecules between the two cell types [3]. Pericytes and ECs together create and maintain the shared basement membrane, the acellular component of the vessel wall [4]. This relationship allows pericytes to regulate the blood flow within vessels by virtue of high levels of α-smooth muscle actin (αSMA) and myosin expression, which can bring about vessel constriction [5]. Pericyte density and the EC to pericyte ratio is found to differ between organs, with ratios estimated to range from 1:1 in the central nervous system (CNS) to 10:1 in tissues such as skeletal muscle [6]. Consequently, in any given organ the proportion of the abluminal vessel surface which is covered by pericytes can be anywhere from 10–70% [7]. At the interface between the endothelial tube and the surrounding tissue, pericytes are ideally located to regulate processes associated with the vasculature, including the control of angiogenesis, which is well documented for these cells both in the context of general homeostasis and in response to trauma. Pericytes mechanically regulate vessel wall integrity, and serve as signalling mediators of EC behaviour. Paracrine pericyte signalling directs EC proliferation and migration to form new vessel sprouts when appropriate and inhibits aberrant pro-angiogenic behaviour in ECs when vessel sprouting is not required [8]. Pericytes have also recently been found to regulate the diffusion of cells and proteins from the vessel to the surrounding tissue, influencing the infiltration of neutrophils [9,10] and macrophages [11], which suggests an additional role for pericytes as mediators of the inflammatory process.

### 1.2. Pericyte Origin and MSC Properties

The developmental origins of the pericyte across all tissues are still not fully understood. For the most part, pericytes develop from the mesoderm during embryogenesis, with the origins of pericytes in the gut [12], lungs [13] and liver [14] having been tracked to the mesothelium. Similarly, cardiac pericytes have been shown to stem from the epicardial mesothelium [15,16]. In the central nervous system, however, chick-chimera studies show that while pericytes in the spinal cord and brain stem have developed from the mesoderm, pericytes of the forebrain are more likely to be derived from neural crest cells of the neuroectoderm [17].

The diverse origins of tissue-specific pericytes are reflected in the antigenic heterogeneity of pericytes observed between tissues. Currently, there are no markers identified as being expressed exclusively by pericytes, nor any constitutively expressed across pericytes of all locations. Within varying tissues, pericytes are found to display morphological changes and differential expression of markers dependent on their differentiation state and specific function within that tissue [18]. Changes in expression are also noted between different developmental stages and disease states [7]. Further, some markers are only expressed when pericytes are actively involved in remodelling of the vasculature, such as RGS5, which is expressed on activated pericytes in tumour development and vascular remodelling but is absent at other times [2,19]. While the list of recognised pericyte markers is growing, there remains a distinct absence within the field of a method by which pericytes can be identified indiscriminately of tissue, disease or developmental factors. As such, pericyte identification still relies on the concurrent identification of perivascular location, morphology and expression of multiple markers. For example, pericytes express many of the same markers as fibroblasts and exhibit similar morphology, so colocalisation between blood vessels and pericytes is often necessary to distinguish between the two cell types. The current struggles in pericyte identification and therefore isolation have been comprehensively reviewed in recent years [7,20,21]. Ansell and Izita discuss the difficulties encountered when comparing previous studies of pericyte function, particularly in vitro, and identify the potential for confounding results due to the unintentional selection of different pericyte subtypes for inclusion in experimental studies [22]. Much of the discussion of pericytes in the literature today addresses the current limitations in our ability to accurately define and isolate pericyte subtypes for experimental purposes.

There is significant overlap between markers expressed by pericytes and mesenchymal stem cells (MSCs), which is perhaps unsurprising given the predominately mesenchymal origin of many pericyte populations. Expression of CD105, CD73, CD90 and CD44 is observed in both pericytes and MSCs [23], and the observation that some subsets of pericytes express αSMA while others do not leads some researchers to postulate that αSMA^+^ pericytes are more likely to carry out a structural support role at the blood vessel wall while αSMA^−^ pericytes possess a more regenerative MSC-like phenotype [24].

Much of the current research in regenerative medicine is invested in evaluating the potential of cells with MSC-like properties as treatments to improve wound healing. We now understand that pericytes not only express MSC markers but also possess MSC-like properties, and display the ability to differentiate in vitro into an array of mesenchymal cell types. These include adipocytes [25], osteoblasts, chondrocytes [26], phagocytes and granulocytes [2]. The potential for pericytes to differentiate into beneficial cell types during the proliferative and regenerative stages of wound healing is an exciting prospect, and in the context of wound healing a cell type with the potential to positively contribute to direct regeneration of lost tissue represents a possible target for therapeutics or a source for the development of a cell-based therapy. Prior to the consideration of these cells for application in cell therapies however, the differentiation potential of all pericyte populations must be comprehensively understood.

Pericyte differentiation potential is extensive and highly dependent on lineage and the surrounding environment [27]. In fact there is a body of observations surrounding pericyte plasticity which, in conjunction with a shared perivascular location, suggests that MSCs and pericytes are, in fact, the same cell type [28]. Recently however, only CD146^+^ bone marrow MSCs (BM-MSCs) and pericytes (also CD146^+^) were found to maintain endothelial tube networks and improve angiogenic sprouting in vitro, while CD146^−^ subtypes of the BM-MSC population did not, suggesting that pericytes are perhaps a subset of MSCs with vascular biology functions which not all MSCs possess [29]. One school of thought with regards to the difficulty of defining the difference between a pericyte and an MSC suggests that a pericyte which is in direct contact through gap junctions with ECs should be termed a pericyte, but that upon liberation of a pericyte from the vessel wall, that same cell should then be termed an MSC [30].

## 2. Pericytes in Wound Healing

Wound healing is a complex process made up of a series of overlapping events that include inflammation, matrix formation and remodelling. Further to our initial understanding of their involvement in the stabilisation of blood vessels and the control of blood pressure, the number of recognised functions of pericytes has broadened drastically, with many of these functions involved in wound healing (Table 1).

### 2.1. Pericytes and Inflammation

Inflammation is one of the key phases during wound healing, and any perturbation in this carefully controlled process can lead to delayed healing, fibrosis or the incomplete healing seen in chronic wounds. It is triggered within 30–40 min of wounding and begins with the influx of neutrophils from the blood vessels to the wound site. Once in the wound, neutrophils act to phagocytose invading pathogens and cellular debris to clear the wound of infection. Early studies of pericytes in inflammation have noted that these cells form umbrella-like covers over gaps between ECs following histamine treatment, which prevents cells and proteins from leaving the vessel. Interestingly, the opposite is seen after IL-2 treatment, which causes pericytes to realign at EC junctions and results in leaky microvessels [34,35]. Neng et al. showed that pericytes also regulate tight junctions in a paracrine manner in EC monolayers in the mouse ear. This was shown to in turn regulate EC monolayer permeability and to control the diffusion of proteins and cells out of the blood [36]. These studies suggest that pericytes play a significant role in controlling inflammation. More recently, pericytes have been shown to be directly involved in the extravasion of neutrophils from the vessels. Direct contact between neutrophils and pericytes induces a relaxation of the pericyte cytoskeleton via the inhibition of RhoA/ROCK signalling, which allows neutrophils to leave the vessel at regions displaying low expression of matrix proteins, termed “low expression regions” (LERs) [10]. This process is facilitated by the expression of Intercellular Adhesion Molecule-1 (ICAM-1), Macrophage antigen-1 (Mac-1) and Leukocyte function associated antigen-1 (LFA-1) [9]. ICAM-1 expression by pericytes, in conjunction with the expression of chemoattractant MIF, has also been shown not only to attract and activate neutrophils and macrophages, but also to facilitate efficient trafficking of these cells to areas of infection [38]. Pericytes have also been shown to influence T cell activity: brain pericytes are able to present antigens to T cells in order to induce lymphocyte activation [39], whereas retinal pericytes inhibit T cell action [40]. Whether these inherent differences in action are due to differences in the local population of pericytes or a result of microenvironmental cues is unclear. Brain pericytes also respond to inflammatory signals, such as lipopolysaccharides (LPS), resulting in activated NF-κB and expression of Interferon gamma-induced protein 10 (IP-10) and Monocyte Chemoattractant Protein-1 (MCP-1) [41]. Blockade of pericyte recruitment to vessels and therefore pericyte-EC intractions induces inflammatory reactions in ECs and results in increased extravasion of macrophages in an adult mouse model of diabetic retinopathy, again illustrating the importance of pericyte influence in the correct regulation of inflammatory infiltration [37]. Hung et al. suggest a role for pericytes not only in the recruitment of immune cells but also in the direct detection of proinflammatory molecules during infection, and show that decreasing the presence of pericyte-like cells in a model of lung injury leads to decreased inflammatory response to infection, leading the authors to propose that pericytes be considered “interstitial immune sentinel cells” [42]. Together, these studies intimately link pericyte action with regulation of the inflammatory response.

### 2.2. Pericytes and Re-Epithelialisation

Another key process during wound healing is the reformation of the epithelial barrier post-wounding. This helps to prevent wound infection and begins to restore some of the vital functions of the skin, such as the prevention of excess water loss and the regulation of temperature. Pericytes have been implicated in this process, and this action is quite distinct from pericyte action at the surface of the vessels. Paquet-Fifield et al. isolated pericytes from skin by means of FACS sorting with a pericyte specific antibody, and created organotypic cultures (OCs), with or without pericytes, which also contained fibroblasts and keratinocytes. When pericytes were present in the OCs there was a drastic improvement in the epidermal layer formed: the epidermis in these OCs was multilayered and sustainable for much greater periods of time when compared to the epidermis of OCs which did not contain pericytes [43].

### 2.3. Pericytes and Angiogenesis

Pericytes have been known to play a role in blood vessel formation from some of the earliest studies of their function, which identified these cells as being distinct from endothelial cells and originally labelled them Rouget cells [47]. More recently, they have been shown to respond to platelet derived growth factor β (PDGFβ) and transforming growth factor β (TGF-β) released by platelets upon injury [2]. The chemotactic response of pericytes to PDGFβ causes these cells to leave the outer layers of blood vessels and migrate into the wound site. This was established by Rajkumar et al. in studies using the PDGFβ inhibitor imatinib [48]. This migration allows ECs to proliferate into the wound site in response to vascular endothelial growth factor (VEGF), which is also released upon platelet activation [49]. This process is aided by the production of fibronectin, vitronectin and laminins, which provide a structure to support EC migration and capillary tube formation [50,51]. The provisional matrix, formed by these ECM components, is frequently remodelled during healing by proteases released by macrophages [52]. This can expose matricryptic sites such as Arg-Gly-Asp (RGD), which act as adhesion sites for EC receptors and can, therefore, regulate EC migration, proliferation and survival [52]. Interestingly, pericytes may regulate protease action via their expression of tissue inhibitor of metalloproteinase-3 (TIMP-3), which prevents capillary tube regression normally caused by matrix metalloproteinase-1 (MMP-1) and -10 (MMP-10) [31]. Pericytes also act to stabilise newly formed capillaries by the expression of TGF-β [32] and by Rho GTPase regulated alterations in pericyte contractility, which inhibit EC proliferation [33]. PDGFβ also appears to be essential in this process, as PDGFβ and PDGFRβ knockout mice exhibit endothelial hyperplasia with a distinct lack of pericytes present on blood vessels [53]. Interestingly, control of PDGFβ expression appears to be via Tie2 and Ang1/Ang2 interactions, where Tie2/Ang1 interaction leads to PDGFβ expression and pericyte recruitment, while Tie2/Ang2 interaction leads to the opposite [54]. In addition to PDGFβ and PDGFRβ, the PDGFβ retention motif is also crucial for pericyte-EC signalling. This sequence of amino acids acts to hold PDGF in close proximity to the EC for it to be recognised by the PDGFRβ expressing pericytes. This allows direct pericyte-EC interaction, as well as creating a PDGF gradient which enables pericytes to migrate to ECs [55]. This motif has been studied using a *pdgf-b^ret/ret^* mouse knockout model, and has been shown to be crucial for maintaining vascular function in the retina, brain and liver [56,57,58].

### 2.4. Pericytes and Matrix Deposition/Fibrosis

Under normal conditions, matrix deposition is initiated once the wound has been cleared of infection and cellular debris. The main cell type responsible for this is the fibroblast. These cells initially deposit fibronectin and collagen III, but in later phases replace these proteins with collagen I and elastin. Fibroblasts, like pericytes, are attracted to the wound site by the expression of PDGF by resident cells and platelets [48]. Once in the wound, fibroblasts may become activated to differentiate into myofibroblasts, expressing α-SMA to physically contract the wound [59]. Interestingly, pericytes are also able to produce collagen [44,45]. The pericytes in these studies appear to remain as collagen secreting cells and don’t express αSMA, suggesting that they do not convert to myofibroblasts unlike the resident fibroblasts within the wound. In an interesting study, Dulauroy et al. were able to use a Cre-transgenic mouse to label ADAM12, which is induced only during embryogenesis and fibrosis. They showed that the majority of collagen producing cells were positive for αSMA and thus were myofibroblasts. These perivascular cells were also shown to be positive for PDGFRβ and NG2, and were presumed to be pericytes [60]. In other studies, pericytes have been shown to differentiate into myofibroblasts to promote fibrosis, particularly in the kidneys where the pericytes present are called mesangial cells [46]. Interestingly, deletion of pericytes does not alter the recruitment of myofibroblasts or alter kidney fibrosis, which suggests that resident MSCs may also play a role in promoting fibrosis, and lends credence to the theory that pericytes are derived from MSC populations rather than the reverse [61]. Birbrair et al. suggest that pericytes could be split into two subsets dependent on their expression of Nestin (type-1: Nestin^−^NG2^+^ and type-2: Nestin^+^NG2^+^). They find that type-1 pericytes accumulate near sites of fibrosis but are not solely responsible for the resultant fibrosis, whereas type-2 pericytes appear to play a role in angiogenesis [62,63]. Pericytes have also been show to play a significant role in fibrosis in the liver as hepatic stellate cells. Mederacke et al. use a Cre-transgenic mouse that marks all stellate cells to show that 82–96% of myofibroblasts in a model of toxic, cholestatic and fatty liver disease are of stellate origin [64]. These studies illustrate that pericytes have a major role in important matrix deposition, but under negative circumstances may promote fibrosis.

Clearly, pericytes can influence each phase of the wound healing process (Table 1), and as such should be considered a major cell type that can regulate healing. With increasing evidence that pericytes can promote fibrosis, these cells may not only be a potential target for therapies to accelerate healing but also to prevent fibrosis. Many of the beneficial aspects of pericytes are due to their plasticity and ability to act in a stem cell-like manner to regulate the microenvironment, resulting in improved healing.

## 3. Pericytes in Other Pathologies

Pericytes mediate both angiogenesis and vessel permeability, consequently they are important in the development of solid tumours, which rely on sufficient vascularization and therefore blood supply to grow.

Pericyte stabilization of the vessel wall supports vascularization within a tumour and can prevent the passing of cancer cell-targeting drugs such as chemotherapeutic agents from the blood stream to the tumour tissue [65]. Consequently, there has been some anti-angiogenic targeting of pericytes within tumours, with a view to destabilizing the vessels that feed tumours and increasing the permeability of cancer drugs into the tumour. Under normal circumstances, however, pericyte signaling represents a fine balance between pro- and anti-angiogenic activities, as pericyte presence not only stabilizes the function of preexisting vessels but also prevents the aberrant proliferation of ECs to form new vasculature. As such, insufficient pericyte coverage in tumours can also be detrimental, resulting in excessive vascular sprouting and increased vascularization of tumours. This suggests that pro-angiogenic targeting of pericytes in tumours may also be beneficial. Additionally, pericyte-EC cross-talk and the resultant regulation of ECs in tumours limits the metastasis of cancer cells, and depleted pericyte coverage of vessels in PDGFβ-deficient mice leads to increased metastasis of solid tumours [66]. Research into the targeting of pericytes in cancer aims to balance pro- and anti-angiogenic signaling to achieve ‘vascular normalisation’ within the tumour microenvironment. Interestingly the neuron-glial antigen 2 (NG2) proteoglycan, which is often used as a pericyte marker in conjunction with the expression of other proteins, appears to play an important role in pericyte biology within the context of tumour angiogenesis. Altered vascularity, including vessel leakiness and decreased pericyte coverage, is noted in models of brain and breast cancer in NG2 null mice [67,68], and this is thought to be the result of decreased pericyte-EC crosstalk. NG2 knockout in a mouse brain melanoma model also appears to decrease tumour blood supply and increase hypoxia, hinting at a possible therapeutic pathway for the treatment of this disease [69,70]. The result of pericyte perturbation in the context of tumour growth however is complex and multifaceted, directly reflecting the delicate nature of normal angiogenic control, and as such the development of pericyte-targeted therapeutics for cancer is difficult.

Pericyte numbers decline in the dermal and muscle capillary networks of diabetic patients, where they also exhibit an altered morphology with hypertrophy, abnormal cytoplasmic branching and gaps in the basement membrane [71]. These pericytes also appear to promote a fibrotic or sclerotic vessel [72,73]. A study which investigated pericyte changes in patients with chronic venous insufficiency found that 31 out of 42 patients displayed an altered pericyte phenotype [74]. Significantly diminished pericyte coverage is also observed on blood vessels in the retinas of diabetic patients experiencing retinopathy, and in fact this is identified as one of the main mechanisms of disease progression [75]. Hyperglycemia in these patients has been shown both in vivo and in vitro to cause pericyte apoptosis leading to increased production of acellular capillaries in the retina [76]. Mechanistic studies identify activation of NF-κB, PKCδ and SHP-1 as effectors in this outcome [76,77]. 

Given that one of the most common diabetic pathologies is angiogenic dysregulation, pericyte dysfunction is not a surprising observation in diabetic patients and models, and indicates the normalisation of pericyte number and function as a promising therapeutic approach for the treatment of diabetic complications.

In humans, a two-fold increase in the pericyte number observed on pulmonary arteries is noted in the lungs of patients with pulmonary arterial hypertension (PAH) when compared with the vessels of healthy control samples. Ricard et al. [78] show that these finding are recapitulated in vivo in models of PAH, and show that these additional pericytes serve as a source of smooth muscle-like cells leading to endothelial dysfunction and excessive remodelling of the pulmonary vasculature which is associated with PAH.

Degeneration of pericytes is also observed at the blood brain barrier (BBB) in patients with Alzheimers disease (AD) [79]. This leads to neurodegeneration, caused by vascular breakdown and decreased cerebral blood flow. It is also suggested that pericyte loss further contributes to neurodegeneration by allowing increased permeability of blood vessels, leading to the buildup of damaging molecules such as plasmin, fibrin and thrombin in the brain [81]. Here, in yet another model of disease, the importance of vascular stability and permeabilisation and how these parameters are regulated by pericytes is illustrated once again.

Pericytes have also been implicated in fibrosis of the kidney. Mouse models of kidney fibrosis indicate that collagen-producing myofibroblasts appear to originate from a perivascular location [83], and genetic tagging of pericytes illustrates these cells can gain αSMA expression and differentiate into myofibroblasts [80]. It is suggested that the activation of pericytes in the kidney to differentiate into myofibroblasts not only leads to fibrosis but also leaves the endothelium of capillaries unstable, leading to the decreased renal vascularization which is documented in chronic kidney disease [83]. With investigations into pericyte action, uncovering the greater role that pericytes play in many divergent pathologies (Table 2) increases the possibilities for future therapeutic treatments targeting pericytes.

## 4. Pericytes as a Therapeutic Agent

With an ever-growing understanding of pericyte potential and function comes new possibilities in the form of pericyte-based therapies. In comparison to the significant momentum that MSC application has gained in recent years, the therapeutic potential of pericytes is under-investigated, however early preclinical studies in mice indicate that pericytes can contribute positively to healing in several different tissues.

The myogenic potential of in vitro pericytes is recapitulated by in vivo studies of pericyte function in mouse models of muscle damage. Human pericytes isolated by expression of CD146, NG2 and PDGFRβ from both skeletal muscle and nonmuscular tissues produce myofibres and contribute to muscle regeneration when injected into a cardiotoxin-induced mouse model of muscle damage [84]. Similarly, cells positive for pericyte markers CD146, NG2 and PDGFRβ isolated from the culture of human pluripotent stem cells (hPSCs) promote muscle regeneration and increased vascularization when applied to a mouse model of limb ischemia. At 21 days, these transplanted cells are found incorporated into both the vasculature and the muscle tissue of the recovering limb [85]. In another study, human placental cells isolated with a CD146^+^CD34^−^CD45^−^CD56^−^ expression profile are shown to produce myofibres and promote increased angiogenesis in the muscles of SCID/*mdx* mice [86]. With their myogenic abilities confirmed in vivo, the development of a pericyte-based therapy holds great potential to enhance the healing of muscles.

Chen et al. have also investigated the therapeutic potential of CD146^+^CD34^−^CD45^−^CD56^−^ pericytes on the ischemic heart. In their study, pericyte application to a mouse model of myocardial infarction (MI) resulted in improved cardiac recovery and contractility, as well as decreased fibrosis and decreased infiltration of inflammatory cell types [11]. The authors report superior recovery following pericyte transplantation when compared to transplantation of CD56^+^ myogenic progenitor cells, which suggests that the positive outcome was not solely due to the myogenic capabilities of pericytes but rather a cumulative effect of pericyte function which may also include regulation of angiogenesis and inflammation. This study also suggests an anti-fibrotic function for pericytes, in contrast to other studies which identify pericytes as significant contributors to fibrosis by way of differentiation into myofibroblasts [87,88,89]. It is possible that in this model the tissue signalling environment did not induce pericyte differentiation into myofibroblasts, and this highlights the fact that pericyte function and heterogeneity are heavily influenced not only by pericyte origin but also by the surrounding tissue environment.

Pericytes (CD146^+^NG2^+^CD45^−^) isolated from mouse fat tissue display osteogenic potential in vitro which is mirrored in vivo by contributing to the regeneration of mouse bone injury when applied in a seeded scaffold [26]. Similarly, human adipose derived pericytes (CD146^+^CD34^−^CD45^−^CD31^−^) enhance bone healing and encourage bone-union in mouse bone fractures with equal efficacy to BM-MSCs [90]. Given this, the authors suggest that adipose derived pericytes present a more preferable option for transplantation than BM-MSCs as they can be isolated abundantly from fat tissue such as that resulting from liposuction and represent a more defined and homogenous population that BM-MSCs.

In the diabetic retina, there is a loss of pericytes which leads to collapse of the vasculature and ultimately blindness. Human cells derived from adipose stem cells and expressing αSMA, PDGFRβ and NG2 can protect against diabetic retinopathy in a mouse model, causing revascularisation of the retina after injection which was not achieved by injection of human BM-MSCs [91]. This effect of pericyte injection was enhanced when the cells were pre-treated with TGF-β1. These results indicate that replacement of lost endogenous pericytes in a diabetic setting can encourage angiogenesis and vascular support, as is seen in acute models of injury, and are particularly promising when considering the possibility that pericyte therapies may hold for treating diabetic pathologies associated with pericyte loss.

Human umbilical cord perivascular cells (HUCPV) isolated by expression pattern CD45^−^CD34^−^SH2^+^SH3^+^Thy-1^+^CD44^+^ and also expressing the pericyte marker 3G5 cause enhanced healing at 3 and 7 days when applied in a fibrin gel to full thickness skin defects in Balb/C mice, as assessed by dermal thickness and re-epithelialisation [92]. In another study, the application of human adipose derived stem cells expressing pericyte markers αSMA, PDGFRβ, NG2 and Ang1 in a PEG-fibrin gel to a rat model of excisional wounding resulted in earlier collagen deposition and remodelling, as well as increased angiogenesis [93]. Contrastingly, the application of human neonatal foreskin dermal pericytes (CD45^−^VLA-1/α1/CD49a^bright^) to a mouse model of excisional wound healing did not enhance re-epithelialisation and in fact resulted in a decrease in dermal wound closure at day 7 when compared to wounds treated with CD45^−^VLA-1^dim^ fibroblasts [94]. The authors suggest that as this model assessed the capability of applied human pericytes to influence the migration of endogenous keratinocytes in wounded mouse skin, signalling between the human and murine cells in question may not have been successful. In addition, this study applied cells isolated based on a different expression pattern when compared to other studies investigating the application of pericytes to wounded skin, and serves to further illustrate the heterogeneity of pericytes and how important standardisation of identification and isolation will be before these cells can be considered a realistic source for the development of cell therapies.

Overall, early studies of pericyte application to mouse models of muscle, heart, bone and skin injury show promising signs that these regenerative and plastic cells can positively contribute to healing, but also raise questions as to how the origin of cell isolation and method of delivery can affect the ability of these cells to carry out beneficial functions. Little is known about the effect of pericyte delivery to chronic tissues, including chronic wounds, but the demonstration in other tissues that applying pericytes can encourage enhanced angiogenesis and decreased inflammatory infiltration as well as regenerating lost tissue is promising when considering the treatment of non-healing wounds. The fibrotic activity of the pericyte and how this is influenced by tissue-specific environments remains incompletely understood, and this is an area which would require significant investigation before the use of pericytes as a clinical cell therapy for wound healing could be considered.

## 5. Conclusions

The functions and capabilities of pericytes are impressive and, as yet, incompletely understood. These cells regulate the vasculature and the inflammatory response, and in addition possess MSC-like regenerative qualities. As such, the pericyte is well placed to significantly influence healing outcomes. A decrease in pericytes associated with the vasculature is well documented in the retinas of diabetic patients, and this results in the onset of diabetic retinopathy. Loss of pericytes is also documented in other disease states, and aberrant pericyte function is identified as an important target in the development of cancer therapies. With each observation of pericyte function or dysfunction in the context of new disease environments, the body of knowledge illustrating the importance of pericytes in the regulation of homeostatic and healing processes grows. There has been a lot of interest in the idea of MSC application as a wound therapy, and it is possible that pericytes, which possess both MSC-like behaviours and distinct regulatory roles in angiogenesis and inflammation, may represent another promising cell population for the development of treatments. In fact, recent in vivo studies show that the transplantation of isolated pericytes can positively influence the healing of bone, muscle and skin and can support revascularisation in a mouse model of diabetic retinopathy. It seems that pericytes have an important part to play in chronic and acute healing processes, and must be considered a crucial cell type as we continue to work towards a comprehensive understanding of healing processes to better advise the development of effective therapies.

## Figures and Tables

**Table 1:** Pericyte functions and their contributions to wound healing.

Wound Healing Process	Pericyte Functions
Angiogenesis	Structural support of existing blood vessels [19]
	Regulation of EC proliferation and migration to form new vessels [8]
	Prevention of capillary tube regression by TIMP-3 expression [31]
	Stabilisation of newly formed capillaries [32,33]
Inflammation	Regulation of vessel permeability [34,35,36]
	Regulation of neutrophil extravasion [9,10]
	Regulation of macrophage extravasion [11,37]
	Control of leukocyte trafficking [38]
	Control of T cell activation [39,40]
	Response to inflammatory signals [41,42]
Re-epithelialisation	Regulation of keratinocyte migration [43]
Fibrosis	Production of collagen [44,45]
	Differentiation into myofibroblasts [46]
Tissue regeneration	MSC-like properties: differentiation potential includes adipocytes, osteoblasts, chondrocytes, phagocytes and granulocytes [2,25,26]

**Table 2:** Pathologies exhibiting pericyte perturbation and likely outcomes of altered pericyte number or function.

Disorder	Pericyte Aberrance Observed	Pericyte Functions Likely to Impact Disease
Diabetic chronic healing	Decreased pericyte numbers in dermis, pericytes exhibit altered morphology [72,73,74,75]	Angiogenesis-decreased vascularisation
		Vessel permeability-leaky vessels lead to prolonged and uncontrolled inflammation
		Fibrosis-pericytes promote fibrotic vessels
		Stem cell properties-replacement of lost cell/tissue types
Diabetic retinopathy	Decreased pericyte numbers, increased pericyte apoptosis [76]	Angiogenesis-decreased control of endothelial proliferation
		Vessel permeability-leakiness of vessels
Solid tumour	Unknown, however control of angiogenesis has long been recognised as an important target in treatment of solid tumours	Angiogenesis-tumour relies on new vasculature for blood supply
		Endothelial control-metastasis of cancer
		Vessel permeability-ability of chemotherapeutic agents to pass from bloodstream to tumour tissue
Pulmonary arterial hypertension (PAH)	Increased pericyte coverage on pulmonary arteries [78]	Angiogenesis-excessive remodelling of pulmonary vasculature and endothelial dysfunction
Alzheimers (AD)	Degeneration at blood brain barrier (BBB) [79]	Angiogenesis-break down of vessels causes decreased cereberal bloodflow leading to neurodegeneration
		Vessel permeability-accumulation of damaging molecules in the brain
Chronic kidney disease (CKD)	Differentiation of pericytes into myofibroblasts [80]	Fibrosis-pericytes thought to be source of myofibroblasts contributing to excessive fibrotic activity
		Angiogenesis-differentiation of pericytes into myofibroblasts leaves less pericytes to stabilise vasculature
